# Chromosome-Level Assembly and Annotation of the Endangered Red-Wing Fish (*Distoechodon macrophthalmus*)

**DOI:** 10.3390/genes15121536

**Published:** 2024-11-28

**Authors:** Xiangyun Zhu, Yanping Luo, Baoshan Ma, Qi Shen, Xingyu Zheng, Mei Xu, Qiang Sheng, Junjie Wu

**Affiliations:** 1 Yangtze River Fisheries Research Institute, Chinese Academy of Fishery Sciences, Wuhan 430223, China; zxy@yfi.ac.cn (X.Z.); baoshanma@yfi.ac.cn (B.M.); 2 Wuhan Design and Engineering College, Wuhan 430205, China; flower-11022@163.com; 3 School of Life Sciences, Huzhou University, Huzhou 313000, China; 15031797627@163.com (Q.S.); z1791763704@163.com (X.Z.); 4 Yunnan Agricultural Broadcast and Television School, Kunming 650041, China; xumei0510@yeah.net; 5 Yunnan Institute of Fishery Sciences Research, Kunming 650111, China

**Keywords:** *Distoechodon macrophthalmus*, genome assembly, annotation, evolution

## Abstract

**Background/Objectives:** The red-wing fish (*Distoechodon macrophthalmus*), an endangered species native to Yunnan, is endemic to Chenghai Lake. The natural population of this species has suffered a sharp decline due to the invasion of alien fish species. Fortunately, the artificial domestication and reproduction of *D. macrophthalmus* have been successful and this species has become an economic species locally. However, there is still little research on *D. macrophthalmus*. **Methods:** In this study, a high-quality genome of *D. macrophthalmus* was assembled and annotated. The genome was sequenced and assembled using the PacBio platform and Hi-C method. **Results:** The genome size is 1.01 Gb and N50 is 37.99 Mb. The assembled contigs were anchored into 24 chromosomes. BUSCO analysis revealed that the genome assembly has 95.6% gene coverage completeness. A total of 455.62 Mb repeat sequences (48.50% of the assembled genome) and 30,424 protein-coding genes were identified in the genome. **Conclusions:** This study provides essential genomic data for further research on the evolution and conservation of *D. macrophthalmus*. Meanwhile, the high-quality genome assembly also provides insights into the genomic evolution of the genus *Distoechodon*.

## 1. Introduction

*D. macrophthalmus*, belonging to the Actinopterygii class, Cypriniformes order, Cyprinidae family, and *Distoechodon* genus [1], is commonly known as the red-wing fish. This species is restrictedly distributed in Chenghai Lake, located in the central part of Yongsheng County, Lijiang City, Yunnan Province, China. Importantly, Peters established the genus *Distoechodon* (Cypriniformes: Cyprinidae) in 1881 with the type species *D. tumirostris*, and *D. macrophthalmus* was described as a distinct species in 2009 by Zhao et al. [1], which has the diagnostic characters of lateral line scales of 78–85 and the predorsal scales of 34–39. As a new species in the genus *Distoechodon*, *D. macrophthalmus* exhibits similarities to *D. multispinnis*, particularly in terms of having higher lateral line and predorsal scale counts, but has relatively bigger eyes that are easily distinguished. *D. macrophthalmus* is an omnivorous fish that mainly feeds on underwater humus, diatoms, filamentous algae, and debris of higher plants [2]. The abundant and diverse algae in Chenghai Lake provide rich food resources for *D. macrophthalmus*, and *D. macrophthalmus* plays an important role in the water purification of Chenghai Lake, which has great industrial and ecological value.

*D. macrophthalmus* was one of the main indigenous economic fishes in Chenghai Lake, ever accounting for up to 30% of Chenghai’s fishing yield before the 1990s [3]. The shift in agricultural production methods around the lake and the rapid growth of spirulina culture in the lakeside area accelerated the eutrophication of Chenghai Lake, leading to an algal bloom outbreak in winter [4]. Additionally, the invasion of non-native fish species posed serious threats to the indigenous fish species in Chenghai Lake. Invasive species miniaturize the zooplankton community, resulting in the massive reproduction of algae and affecting the water quality. The number of indigenous fish species decreased from fifteen (six endemic) to ten, and the production of indigenous fish decreased sharply [5]. In 2004, no adult specimen of *D. macrophthalmus* was detected in Chenghai Lake and it was inferred that the primary threat stemmed from the introduction of icefishes (Family Salangidae) [1,6]. Impact factors such as overfishing of *D. macrophthalmus* and the invasion of Salangid fish have led to a sharp decline in the yield of *D. macrophthalmus*, accounting for less than 0.2% of the production of Chenghai fisheries [3]. Therefore, the artificial domestication and breeding of this fish have been in progress since 2004. At present, the population of *D. macrophthalmus* has recovered with the success of artificial breeding and release. Nowadays, over 1.2 million fries of *D. macrophthalmus* have been released into Chenghai Lake, greatly alleviating its population resources, protecting the aquatic biodiversity of Chenghai Lake, and promoting the sustainable development of fisheries.

The evolutionary status of *Distoechodon* is still ambiguous and confusing, especially when compared to the most similar genus, *Xenocypris*. In 2021, Liu et al. [7] sequenced the complete mitochondrial genome of *Xenocypris fangi* and found that *X. fangi* was closely related to *D. tumirostris*. In 2022, Zhang et al. [2] compared all the mitochondrial genomes of the *Xenocypris* subfamily and found that the genetic distance between *Distoechodon* and *Xenocypris* is very short. In 2023, Li et al. [8] evaluated the phylogenetic relationship and differentiation time of Xenocyprinae species based on two mitochondrial genes and five nuclear gene sequences and further distinguished different genera. At present, the extant scholarly literature pertaining to *D. macrophthalmus* is notably scarce, with a significant proportion of the available data being derived from reports issued by local governmental authorities within China. In addition, there are no available genomes of *D. macrophthalmus* that have been reported. Therefore, a high-quality reference genome and annotation of *D. macrophthalmus* is essential to reveal the phylogenetic relationships and the unique evolutionary characteristics of the genus *Distoechodon*. This study is the first genome report of *D. macrophthalmus* and offers crucial genomic resources and new perspectives for further genetic breeding and conservation studies on *Distoechodon*.

## 2. Materials and Methods

### 2.1. Sample Collection

**Figure 1:**
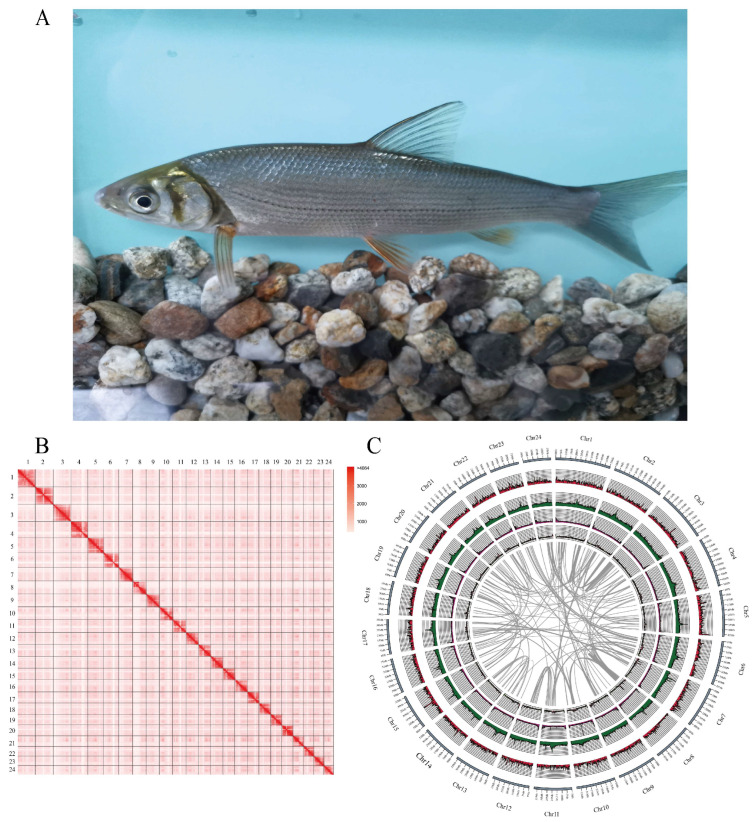
Overview of the *D. macrophthalmus*. (**A**) *D. macrophthalmus* in dorsal view; (**B**) Hi-C interactive heatmap of genome-wide *D. macrophthalmus*. The depth of red color shows the contact density. A square represents a chromosome and the number represents the chromosome ID; (**C**) Circos of *D. macrophthalmus* genome characteristics. From outside to inside: gene density; transposon density; repeat elements; distribution of GC; self-collinearity of genes.

A female individual of *D. macrophthalmus* was collected from Chenghai Lake, Yunnan province (Figure 1A) and the blood tissue was sampled for DNA sequencing. High-quality genomic DNA was extracted by the QIAGEN DNeasy Blood and Tissue Kit (QIAGEN, Shanghai, China) and the DNA quality and quantity were examined using a NanoDrop 2000 spectrophotometer (NanoDrop Technologies, Wilmington, DE, USA), a Qubit 3.0 Fluorometer (Life Technologies, Carlsbad, CA, USA), and electrophoresis on a 0.8% agarose gel, respectively. Total RNA was extracted from five tissues of the specimen, including muscle, liver, brain, blood, and kidney, using Trizol reagent (Invitrogen, CA, USA). RNA purity and integrity were monitored by a NanoDrop 2000 spectrophotometer (NanoDrop Technologies, Wilmington, DE, USA) and an Agilent Bioanalyzer 2100 system (Agilent Technologies, California, USA). Ethics Committee: Animal experimental ethical inspection of laboratory animal center, Yangtze River Fisheries Research Institute, Chinese Academy of Fishery Sciences (Approval code: YFI2021ZXY06; Approval date: 5 December 2021).

### 2.2. Genome Sequencing and RNA-seq

An SMRTbell library was constructed using the SMRT Express Template Prep Kit 2.0 (Pacific Biosciences, California, USA) for sequencing on a PacBio Sequel II system by Frasergen Bioinformatics Co., Ltd. (Wuhan, China). High-quality Circular Consensus Sequencing (CCS) reads were obtained by using the CCS program [9] for preprocessing. The DNA sample extracted from blood tissue was used for the construction of the Hi-C library with a 4-cutter restriction enzyme MboI. The Hi-C library was sequenced using the Illumina HiSeq X platform with 150 bp paired-end mode. An RNA sequencing library was constructed from a pooled sample with the equal amount of RNA extracted from the muscle, liver, brain, blood, and kidney. The full-length cDNA was prepared using a SMARTer™ PCR cDNA Synthesis Kit [10] (Takara Biotechnology, Dalian, China). Subsequently, SMRT sequencing was performed on a PacBio Sequel II platform.

### 2.3. Genome Assembly and Hi-C Scaffolding

Initially, jellyfish [11] was used to calculate the *k*-mer frequency and GCE [12] was used to estimate genome size, heterozygosity, and repetitive sequences. The CCS software v6.4.0 was used to generate the consensus reads (HiFi reads) with the parameter ‘-minPasses 3’. Subsequently, Hifiasm [13] was used to assemble these HiFi reads preliminarily. To identify the association between different contigs, the clean reads generated from the Hi-C library were mapped to the assembled contigs using Juicer [14] with the default parameters. Self-ligation, non-ligation, and other invalid reads were filtered using the Hicup software v0.8.2 [15]. Finally, error correction of the assembly generated by 3d-DNA [16] was performed using the juicebox [17] program to obtain the final chromosome-level genome. In order to assess the integrity of the assembly, the HiFi reads were realigned to the final assembly utilizing minimap2 v2.5 [18], employing the default parameters. To assess the completeness of the genome assembly, a quantitative evaluation was performed using the Benchmarking Universal Single-Copy Orthologs (BUSCO) v3.1 [19] with the actinopterygii_odb9 geneset. We evaluated the mapping rate by mapping the PacBio and Illumina sequencing reads to the assembly using bowtie2 [20] and minimap2. The short reads were *k*-merized using jellyfish (*k*-mer = 21) and then the *k*-mer completeness scores were estimated using Merqury v1.3 [21].

### 2.4. Repeat and Protein-Coding Gene Annotation

The Tandem Repeats Finder v4.09 (TRF) [22] was utilized to predict the tandem repeats in the genome. The identification of repeat contents was achieved through the integration of homology-based predictions and de novo predictions. The known transposable elements (TEs) were identified using RepeatMasker v4.0.9 [23] with the Repbase TE library. Meanwhile, RepeatModeler [24] was used to construct a de novo repeat library. Additionally, we conducted a de novo investigation of long terminal repeat (LTR) retrotransposons within the genomic sequences of *D. macrophthalmus* using LTR_FINDER v1.0.7 [25], LTR_harvest v1.5.11 [26], and LTR_retriever v2.7 [27]. Ultimately, we integrated the library files from both methodologies and employed RepeatMasker v4.0.7 to analyze the repetitive elements present in the data. We predicted the protein-coding genes using three approaches, including ab initio gene prediction, homology-based gene prediction, and RNA-Seq-guided gene prediction. Augustus v3.3.3 [28] and GeneScan were used to perform the ab initio gene prediction. Gene models were developed utilizing a collection of high-quality proteins derived from the RNA-Seq dataset. Maker v2.31.10 [29] was used to conduct the homology-based gene prediction. The homology protein sequences obtained from five closely related species (i.e., *Danio rerio*, *Ctenopharyngodon idella*, *Megalobrama amblycephala*, *Triplophysa tibetana*, and *Colossoma macropomum*) were aligned to the genome assembly. Additionally, the transcripts were obtained from PacBio SMRT reads using the ISO-Seq pipeline [30] and aligned to the genome using PASA [31]. Finally, EVidenceModeler (EVM) v1.1.1 [32] was used to integrate the predictions to obtain the final gene models.

The functional annotations were performed with the public databases, including the non-redundant protein database (NR), Kyoto Encyclopedia of Genes and Genomes (KEGG), Swiss-Prot, TrEMBL, euKaryotic Orthologous Groups (KOG), Gene Ontology (GO), and Pfam databases, using diamond v0.9.30.131 [33] blastp with the parameters “–outfmt 6 –max-target-seqs 1 –evalue 1 × 10^−6^”. Additionally, special functional databases such as the Comprehensive Antibiotic Research Database (CARD), Carbohydrate-Active Enzymes Database (CAZy), Phibase (PHI), and Virulence Factors Database (VFDB) were used to functionally annotate the proteins. Annotations of noncoding RNA, including tRNA, rRNA, miRNA, and snRNA, were also performed. We used tRNAscan-SE v1.3.1 [34] to identify the tRNA; we identified rRNAs using RNammer v1.2 [35] with the parameters “-S euk -m lsu, ssu, tsu”. MicroRNAs and snRNAs were identified by CMSCAN [36] v1.1.2 software against the Rfam v14.0 [37] database with default parameters.

### 2.5. Gene Family and Evolutionary Analysis of D. macrophthalmus

To delineate gene families derived from protein-coding genes, protein sequences from *D. macrophthalmus* and 40 other closely related species were collected. The identification of gene families was performed using OrthoFinder v2.0 [38]. A phylogenetic tree of *D. macrophthalmus* and the 40 other fish species was constructed using the MUSCLE v3.8.31 [39] program and RAxML v8.2.11 [40]. We used CAFÉ v3.1 [41] to analyze gene family expansion and contraction.

To investigate the chromosome evolution of *D. macrophthalmus* and *D. rerio* (Zebrafish), a genome alignment between the *D. macrophthalmus* and Zebrafish genomes was generated using LASTZ v1.1 [42] with the parameter settings “T = 2 C = 2 H = 2000 Y = 3400 L = 6000 K = 2200”. Following the exclusion of aligned blocks shorter than 2 kilobases, the syntenic relationships between the two genomes were illustrated using Circos v0.69-6 [43].

## 3. Results and Discussion

### 3.1. Genome Sequencing and Assembly

The genome size estimated by GCE was 957.64 Mb, the heterozygosity was 0.4%, and the repeat sequence content was 38%. A total of 1,888,999 high-quality ccs reads and 35.36 Gb HiFi bases were generated by PacBio Sequel II systematic sequencing and the N50 was 18,854 bp in length. The ccs reads were assembled into primary contigs using hifiasm, yielding 89 contigs. The genome assembly of *D. macrophthalmus* is 1.01 Gb in size with an N50 length of 36.05 Mb. A total of 95.09 Gb of Hi-C data, corresponding to approximately 99× coverage, was generated using the MGI-seq platform and utilized for the assembly at the chromosome level. Following the quality control assessment of Hi-C reads conducted with Hicup software v0.8.2, the effective data yield was determined to be 28.14% (27.9 Gb, ~28X coverage depth). The assembled chromosome-level genomes contain 29 contigs and 62 scaffolds. Utilizing Hi-C data, a total of 29 contigs were successfully anchored to 24 chromosomes, resulting in an aggregate length of 939.42 Mb. The lengths of the anchored chromosomes varied, ranging from 29.29 Mb to 55.47 Mb (Table 1). Subsequently, the assembled genomes were subjected to BUSCO with the actinopterygii database to evaluate the completeness of the genome. Using the 4584 direct homologous single-copy gene database constructed by BUSCOs as a reference, the assembly of *D. macrophthalmus* included 4385 (95.6%) complete BUSCOs, of which 4228 (92.2%) were complete single-copy BUSCOs and 157 (3.4%) were completely duplicated BUSCOs. The mapping rate of PacBio long reads to the assembly was 99.38% and the mapping rate of short reads to the assembly was 99.86%. The *k*-mer based QV (quality value) was 59.24. These results indicate a high quality of genome assembly in this study (Figure 1B,C).

### 3.2. Genome Annotation

A total of 455.62 Mb tandem repeat sequences were predicted, accounting for approximately 48.50% of the genome. Among them, long terminal repeats (LTRs) accounted for 3.32%, DNA transposons accounted for 3.25%, and long interspersed nuclear elements (LINEs) accounted for 1.81%. Subsequently, we predicted 30,424 protein-coding genes in *D. macrophthalmus* genome. The average length of the protein-coding genes was 14,272 bp, with a GC content of 50.4%, and the completeness of the predicted protein-coding genes as assessed by BUSCO was 91.6%. A total of 30,376 genes (99% of all predicted genes) were annotated by the seven known databases (Table 2). Additionally, special functional databases such as the Comprehensive Antibiotic Research Database (CARD), Carbohydrate-Active Enzymes Database (CAZy), Phibase (PHI), and Virulence Factors Database (VFDB) were used to functionally annotate the proteins and 2168 genes were successfully annotated. Meanwhile, a total of 604 rRNAs, 346 snRNAs, 14,422 tRNAs, and 21 snoRNAs were identified in the genome (Table 3).

### 3.3. Evolutionary Analysis of D. macrophthalmus

**Figure 2:**
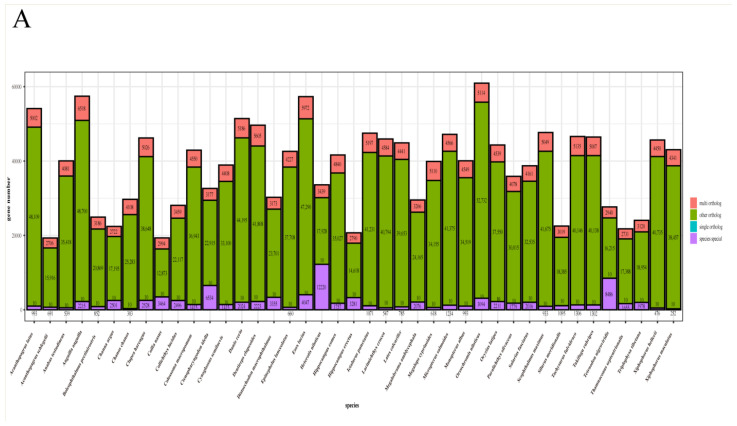

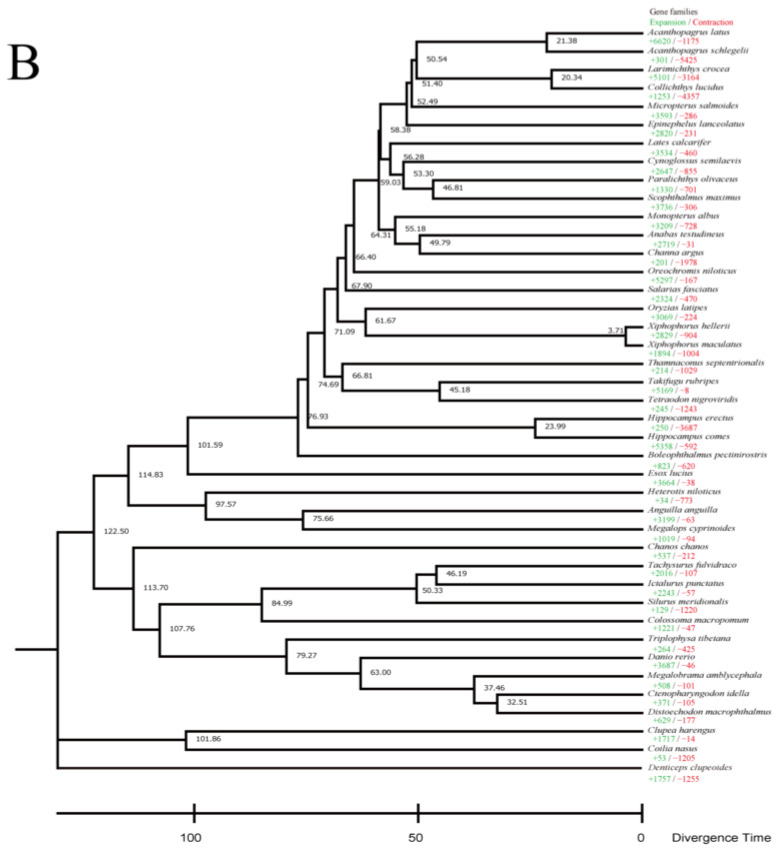
Comparative genomic analysis reveals phylogenetic positioning and genome evolution of *D. macrophthalmus*. (**A**) Statistics of orthologous gene families in 41 representative fish species; (**B**) Phylogenetic tree and estimated divergence time of *D. macrophthalmus* and other representative species, where *D. macrophthalmus* is represented in red font. Statistical analysis of contraction and expansion of gene families.

**Figure 3:**
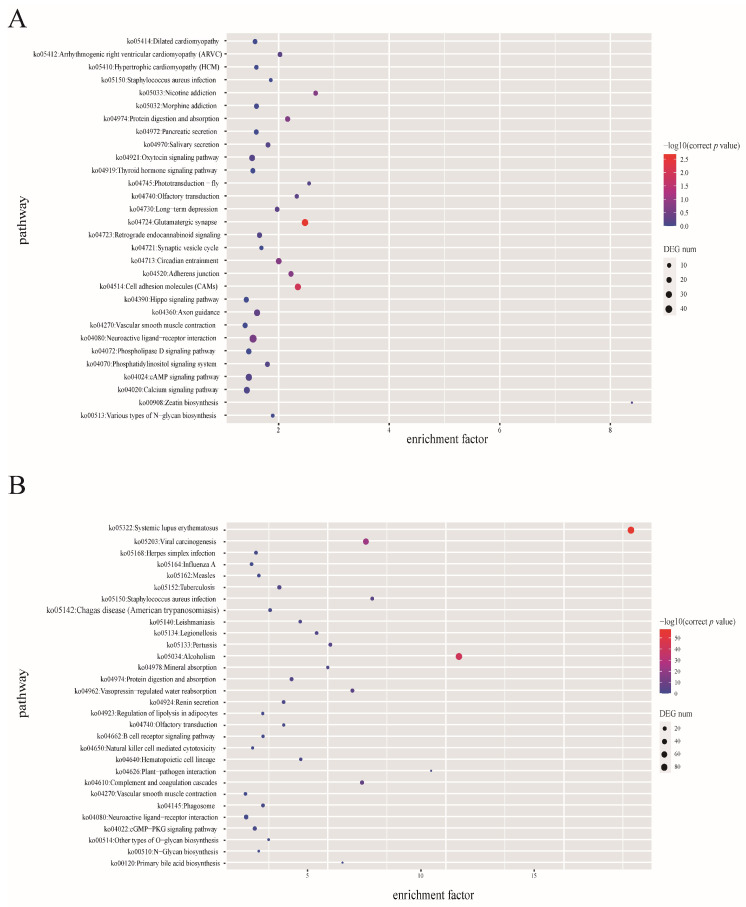
KEGG enrichment analysis of gene families. (**A**) Enrichment analysis of *D. macrophthalmus* specific gene families; (**B**) enrichment analysis of *D. macrophthalmus* expansion gene family.

Based on the protein sequences of *D. macrophthalmus* and 40 other fish species, a total of 1,583,574 gene families were identified from the 41 fish species, of which 2568 genes were shared by the selected species, representing ancestral gene families (Figure 2). Importantly, 3355 gene families were unique to the *D. macrophthalmus* genome. KEGG enrichment analysis of *D. macrophthalmus*-specific genes showed that the specific genes were significantly enriched in environmental information processing, cellular processes, and organismal systems (Figure 3A). The phylogenetic tree showed that *D. macrophthalmus* was grouped with the species in families of Ctenopharyngodon and Megalobrama, indicating a closer relationship with these species. *C. idella* and *D. macrophthalmus* share a common ancestor and the divergence of these two species is at around 32.51 MYA; the divergence time between the first two and *M. amblycephala* was estimated to be 37.46 MYA before (Figure 3B). A prior investigation presents genetic evidence indicating that the divergence of the *Xenocypris* species from the *Distoechodon* and *Pseudobrama* species occurred approximately 10 MYA [2], as determined through mitochondrial gene analysis. The significant divergence among *Xenocyprinae* species is likely to have transpired during the Middle to Late Miocene and Late Pliocene epochs, implying that the processes of speciation and diversification may be linked to the climatic influences of the Asian monsoon.

The expansion or contraction of gene families plays a key role in driving the adaptive evolution of *D. macrophthalmus*. In comparison with gene families of *C. idella*, gene families significantly expanded and contraction increased by 258 and 72, respectively. KEGG enrichment analysis of the expanded gene families demonstrates that they were mainly assigned to “signal transduction”, “signaling molecules and interaction”, “transport and catabolism”, “immune system”, “endocrine system”, “digestive system”, and “sensory system” (Figure 3B). The results of the KEGG enrichment analysis on gene family expansion showed that the expansion gene family enriched in the hematopoietic cell lineage of *D. macrophthalmus* is consistent with the lifestyle of *D. macrophthalmus* living in turbulent rivers in the high-altitude area of Chenghai Lake. Genes related to olfactory perception can increase *D. macrophthalmus*’s adaptability to environmental changes. Therefore, genes related to olfactory translation are significantly expressed in both *D. macrophthalmus’s* unique genes and the expansion gene families.

### 3.4. Synteny of D. macrophthalmus and Zebrafish Genome

**Figure 4:**
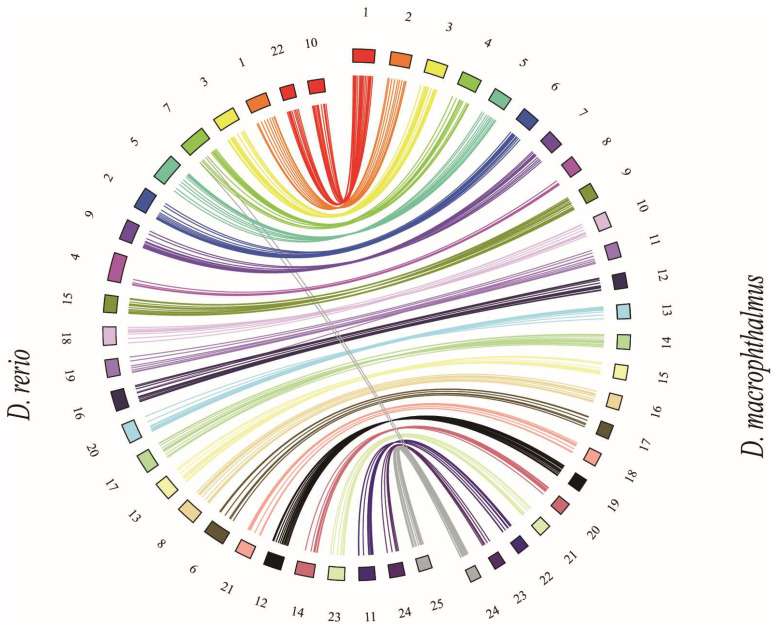
Circos diagram of chromosome synteny between *D. macrophthalmus* and *D. rerio*. Each colored line represents a gene model match between the two species.

In order to conduct a more comprehensive assessment of the quality of genome assembly, the synteny of the *D. macrophthalmus* genome (*n* = 24) with the zebrafish genome (*n* = 25) was investigated. Figure 4 illustrates the gene synteny between the genomes of *D. macrophthalmus* and zebrafish. The chromosomes of *D. macrophthalmus* demonstrated a significant degree of homology with those of the zebrafish, with one chromosome corresponding to zebrafish chromosomes 22 and 10. This indicates that zebrafish possess 25 pairs of chromosomes, whereas *D. macrophthalmus* is characterized by having only 24 pairs of chromosomes. This observation aligns with earlier findings related to grass carp and blunt snout bream [44]. This research provides additional evidence that East Asian cyprinids may exhibit a chromosomal configuration consisting of only 24 pairs, a condition attributed to the fusion of two ancestral chromosomes [45,46]. Research on Drosophila species indicates that chromosome fusion may significantly contribute to adaptive evolution and speciation [47,48,49]. This process can result in reproductive isolation among species, thereby facilitating the emergence of new species.

## 4. Conclusions

In the present study, we have constructed a chromosomal-level genome assembly of *D. macrophthalmus*, which provides a reference for genomic studies of *D. macrophthalmus* in the future. We revealed that a common chromosome fusion event happened in the ancestral East Asian cyprinid. Additionally, the systematic phylogenetic relationships of the East Asian cyprinid were reconstructed, which contributed to a better understanding of the confusing taxonomic relations of the East Asian cyprinid. The expanded gene families characterized an adaptive evolution that could explain the restricted distribution of *D. macrophthalmus*. These genomic data serve as a significant resource for advancing research on economically important *Xenocyprinae* fish species, particularly in the areas of evolution, conservation, and aquaculture breeding.

## Figures and Tables

**Table 1:** Summary of chromosome length of *D. macrophthalmus* genome.

Pseudo-Chromosomes	Length (bp)	Percentage (%)
Chr01	55,467,930	5.50%
Chr02	52,268,502	5.19%
Chr03	52,175,653	5.18%
Chr04	50,776,516	5.04%
Chr05	47,134,997	4.68%
Chr06	45,016,773	4.47%
Chr07	42,254,842	4.19%
Chr08	39,626,809	3.93%
Chr09	39,485,572	3.92%
Chr10	39,347,628	3.90%
Chr11	37,993,288	3.77%
Chr12	37,680,446	3.74%
Chr13	36,358,430	3.61%
Chr14	36,223,197	3.59%
Chr15	36,052,844	3.58%
Chr16	35,777,427	3.55%
Chr17	35,622,143	3.54%
Chr18	34,144,175	3.39%
Chr19	33,406,070	3.32%
Chr20	32,652,807	3.24%
Chr21	32,218,678	3.20%
Chr22	30,659,034	3.04%
Chr23	29,287,022	2.91%
Chr24	27,792,149	2.76%
Unmapped	68,215,872	6.77%
Total	1,007,638,804	100.00%

**Table 2:** Number of genes annotated using different databases.

Database	Number of Annotated Genes	Percentage
GO	14,606	48%
kEGG	13,416	44%
KOG	17,520	57%
NR	30,229	99%
Pfam	22,982	75%
swiss_prot	23,051	75%
TrEMBL	29,350	96%
Total	30,376	99%

**Table 3:** Number of the annotated non-coding RNA.

Type		Number	Total Length	Average Length
rRNA	18s_rRNA	22	41,761	1898.23
	28s_rRNA	21	109,601	5219.10
	5.8S_rRNA	24	3672	153.00
	5S_rRNA	537	62,757	116.87
tRNA		14,422	1,071,936	74.00
snRNA		346	54,608	157.83
snoRNA		21	2707	128.90

## Data Availability

The final genome assembly has been deposited into the Genome Warehouse (GWH) of National Genomics Data Center (NGDC) with the accession number GWHEUUS00000000.1. The Hi-C (SRR28352443), Iso-seq (SRR28352442), and HiFi (SRR28352444) reads have been deposited in the NCBI Sequence Read Archive (SRA) database under BioProject number PRJNA1084914. The genome annotation files have been saved in figshare with the accession number 10.6084/m9.figshare.26310445.
